# Surgical Treatments for Femoral Shaft Fractures: A Narrative Review

**DOI:** 10.5704/MOJ.2311.002

**Published:** 2023-11

**Authors:** BS Fu, ZH Zheng

**Affiliations:** 1 Department of Orthopedic Surgery, Zuoying Branch of Kaohsiung Armed Force General Hospital, Kaohsiung City, Taiwan; 2 Department of Orthopedic Surgery, Hualien Armed Forces General Hospital, Hualien City, Taiwan

**Keywords:** intramedullary nail, malrotation, venous thromboembolism, femoral shaft fracture, nail length

## Abstract

Femoral shaft fractures are increasingly common due to various traumatic injuries. Intramedullary nail (IMN) is considered the gold standard treatment for these fractures, but comorbidities often require thorough trauma life support and intensive care. The primary goal of treatment is rigid fixation, early mobilisation, and long-term functional recovery. This article reviews current concepts in the treatment of femoral shaft fractures, including the effects of early or delayed operation, differences between antegrade or retrograde intramedullary nailing, alternative methods to using a fracture table, methods to predict nail length before operation, assessing femoral rotation during an operation, and complications.

## Introduction

Fractures of the femoral shaft are a common injury treated by orthopaedic surgeons. The incidence of femoral shaft fractures varies from 37 per 100,000 person-years in the United States to 10 per 100,000 person-years in Europe^1,2^. Fractures of the femoral shaft (FSF) tend to occur in two distinct age groups - high energy trauma in the younger population and low energy trauma in the older population. Intramedullary nailing (IMN) is the most common treatment for physiologically stable patients.

The primary goals of treatment are rigid fixation, early mobilisation and long-term functional recovery.

## Materials and Methods

Search strategy: A narrative review of the literature was performed using PubMed-Medline and the Cochrane Database of Systematic Reviews. The query was performed on June 4th, 2023, using the following keywords in combination with Boolean operators AND or OR for the literature search: “intramedullary nail,” “femoral shaft fracture,” “malrotation,” “venous thromboembolism,” nail length,” “complications”. Inclusion criteria were: human clinical trials (prospective and retrospective), systemic review, meta-analysis presented in the English language.

On the other hand, basic science articles, editorials, surveys, special topics, letters to the editor, personal correspondence and review articles, and studies reporting were excluded from the present study.

### Operative Strategy for Femoral Shaft Fractures

The timing of internal fixation for femoral shaft fractures (FSF) in trauma patients and its impact on complications have been the subject of much attention over the past 20 years. Early stabilisation has been shown to benefit patients, and the concept of early total care was introduced^3^. More recently, there has been a shift from damage control orthopaedics to early definitive care, with fixation within 24 hours of presentation being recommended for resuscitated patients^4^.

A study by Obey *et al*^5^ found that delayed fixation of FSF (>48 hours) was associated with higher mortality rates (OR 3.60; CI, 3.13–4.14), longer hospital length of stay (OR 2.14; CI, 2.06–2.22), longer ICU length of stay (OR 3.92; CI, 3.66–4.20), more days on a ventilator (OR 5.38; CI, 4.89– 5.91), and more post-operative complications (OR 2.05; P<0.0001) in patients who underwent fixation. The authors recommend early evaluation of surgical candidacy and preoperative medical optimisation for patients with FSF, with the goal of achieving early fixation (<24 hours of presentation) whenever possible.

Joon-Woo Kim and colleagues^6^ found no significant difference in malalignment, Knee Society scores, union rate, or union time between antegrade and retrograde intramedullary nailing (IMN) for femoral subtrochanteric fractures (FSF). Amit Davidson and colleagues^7^ also found no significant difference in infection, functional outcomes, non-union, and re-operations between antegrade and retrograde IMN in the treatment of severe open femoral shaft fractures.

According to Karpos *et al*^8^, their experience suggests that intramedullary (IM) nailing of acute femoral shaft fractures (FSF) using only manual traction is a safe and effective alternative to using a fracture table. This technique is particularly useful in situations where there are associated unstable spine or pelvis fractures, multiple injury patients, or bilateral femoral shaft injuries.

In their study, Pearson *et al*^9^ presented a method for predicting the length of an antegrade or retrograde intramedullary nail (IMN) prior to surgery. Specifically, the authors found that antegrade nail length could be predicted using the formula: 178 x (height in meters) + 85, with a correlation coefficient of 0.55 (p < 0.01) and an accuracy of 88%. Similarly, retrograde nail length could be predicted using the formula: 198 x (height in meters) + 27, also with a correlation coefficient of 0.55 (p<0.01) and an accuracy of 88%. For example, a patient with a height of 182cm would have a predicted antegrade nail length of 178 x 1.82 + 85 = 408.96mm. The study included patients with heights ranging from 1.17 meters to 2.08 meters and nail lengths ranging from 300-480 mm. The authors found that there was a significant correlation (p<0.01) between patient height and optimal nail length for both antegrade and retrograde IMN procedures.

A study by Marchand *et al*^10^ presented a technique for evaluating femoral rotation during surgery. The lesser trochanter profile was found to be reliable for this purpose. Using this technique, surgeons can detect and correct malrotation of the femoral shaft up to 20° during the operation. The technique involves obtaining a true lateral view of the distal femoral condyles to control rotation. Next, the image intensifier is rotated by 90° to obtain a second view of the proximal femur, which includes the lesser trochanter. The study found that the size of the lesser trochanter on the image was significantly different when there was 15° of malrotation.

### Complications

A prospective cohort study conducted by Patel *et al*^11^ followed 417 patients over a 10-year period to identify risk factors for femoral malrotation after intramedullary nailing resulting from gunshot wounds. The study found that the following risk factors were not associated with femoral malrotation: time of surgery, fellowship training, BMI, and rod entry site. However, the Winquist classification for femoral shaft fractures 3 (comminuted with a large butterfly fragment greater than 50% of the bone's width) and 4 (severe comminution of an entire segment of bone) were linked to femoral malrotation. The average post-operative rotational deformity was 16°.

In a study by Patch *et al*^12^, it was found that the clinical outcomes of femoral shaft fractures caused by firearms were less favourable compared to blunt injuries. The study revealed a significant difference between the two groups in terms of hospital length of stay, thigh compartment syndrome, and the need for soft tissue reconstruction. Thus, physicians should give importance to the development of thigh compartment syndrome.

According to Lin *et al*^13^, poor prognostic indicators in complex fractures of the femoral shaft include bony fragments displaced more than 10mm and reversed wedge bony fragments. These indicators suggest less favourable outcomes. In addition, the union rate 12 months post-operation for small butterfly fragments (mean size of 64.7mm) was 76%, while the union rate for large butterfly fragments (mean size of 78.5mm) was 21%.

### Combined Procedures and Prophylactic Medications

The study conducted by Hall *et al*^14^ aimed to compare embolic phenomena between reamer-irrigator-aspirator (RIA) and standard reamers. The authors quantified the embolic load by performing a transoesophageal echocardiogram during nailing procedures. They found no significant difference in gas data and embolism between the two reaming techniques.

According to a study conducted by Lowe *et al*^15^, the incidence rates of venous thromboembolism (VTE) in different types of orthopaedic surgeries were as follows: pelvis ORIF 1.70%, acetabulum ORIF 0.42%, femoral neck ORIF 0.98%, intertrochanteric ORIF 0.59%, femoral IMN 1.33%, and tibial IMN 0.34%. The study found that pelvis ORIF and femoral IMN had higher incidence rates of VTE, while intertrochanteric ORIF had lower rates.

The Eastern Association of Trauma Surgery (EAST)^16^ and the American College of Chest Physicians (ACCP)^17^ have issued recommendations for preventing VTE. However, there still exists significant variation in VTE prophylaxis within orthopaedic surgery.

We cannot draw conclusions on chemoprophylaxis protocols that include factor Xa inhibitors or aspirin. Although aspirin is approved by the American College of Chest Physicians (ACCP) for treating hip joint fractures, neither aspirin nor factor Xa inhibitors are approved or routinely used for general use in traumatic settings. Further studies are needed to determine whether factor Xa inhibitor regimens are safe and effective for preventing VTE.

## Results

The study suggests that operating on a femoral shaft fracture as soon as possible is advised, as delayed treatment beyond 48 hours results in less favourable outcomes. Both antegrade and retrograde nailing techniques have similar clinical outcomes, and the choice of technique depends on physician preference. During intramedullary nailing, it is crucial to assess femoral rotation to prevent malrotation, and using image intensifiers with a lesser trochanter profile is recommended for this purpose.

This article also highlights the importance of venous thromboembolism (VTE) prophylaxis, especially for patients with certain risk factors such as incomplete prophylaxis, a prior history of VTE, coagulopathy, or concomitant lower extremity fractures. The article underscores the need for further study to determine the use of reamer-irrigator-aspirator (RIA) in intra-operative procedures.

Overall, the article provides a comprehensive overview of surgical treatment options for femoral shaft fractures, addressing various aspects including treatment concepts, surgical techniques, complications, and the importance of VTE prophylaxis.

## Conclusion

It is advised that a femoral shaft fracture should be operated on as soon as possible, as delayed treatment for over 48 hours results in less favourable outcomes for patients. Antegrade or retrograde nailing has similar clinical outcomes, the decision on which type of internal fixation to use was based on physician preference. During intramedullary nailing for femoral shaft fracture, it is critical to assess femoral rotation by image intensifier with a lesser trochanter profile to prevent malrotation. When treating complex femoral shaft fracture, it is critical that bony fragments are approximated. According to our knowledge, we cannot recommend the use of RIA in intra-operative procedures and further study is warranted. Although aspirin or factor Xa inhibitors are not approved or routinely used for general use in traumatic settings, it is still critical to ensure VTE prophylaxis. This is especially important for patients with incomplete prophylaxis, a prior history of VTE, coagulopathy, or concomitant lower extremity fractures.
